# Resistance and resilience to Alzheimer’s disease pathology are associated with reduced cortical pTau and absence of limbic-predominant age-related TDP-43 encephalopathy in a community-based cohort

**DOI:** 10.1186/s40478-019-0743-1

**Published:** 2019-06-07

**Authors:** Caitlin S. Latimer, Bridget T. Burke, Nicole F. Liachko, Heather N. Currey, Mitchell D. Kilgore, Laura E. Gibbons, Jonathan Henriksen, Martin Darvas, Kimiko Domoto-Reilly, Suman Jayadev, Tom J. Grabowski, Paul K. Crane, Eric B. Larson, Brian C. Kraemer, Thomas D. Bird, C. Dirk Keene

**Affiliations:** 1 Division of Neuropathology, Department of Pathology, University of Washington, Seattle, WA 98104 USA; 2 Kaiser Permanente Washington Health Research Institute, Seattle, WA USA; 3 Geriatrics Research Education and Clinical Center, Veterans Affairs Puget Sound Health Care System, Seattle, WA USA; 4 Division of Gerontology and Geriatric Medicine, Department of Medicine, University of Washington, Seattle, WA USA; 5 Department of Medicine, University of Washington, Seattle, WA USA; 6 Department of Neurology, University of Washington, Seattle, Washington USA; 7 Deparment of Radiology, University of Washington, Seattle, Washington USA; 8 Department of Psychiatry and Behavioral Sciences, University of Washington, Seattle, Washington USA; 9 Division of Medical Genetics, Department of Medicine, University of Washington, Seattle, WA USA

**Keywords:** Resistance, Resilience, Alzheimer’s disease neuropathologic change, TDP-43, Hyperphosphorylated tau, Dementia, *C. elegans*

## Abstract

Alzheimer’s disease neuropathologic change (ADNC) is defined by progressive accumulation of β-amyloid plaques and hyperphosphorylated tau (pTau) neurofibrillary tangles across diverse regions of brain. Non-demented individuals who reach advanced age without significant ADNC are considered to be resistant to AD, while those burdened with ADNC are considered to be resilient. Understanding mechanisms underlying ADNC resistance and resilience may provide important clues to treating and/or preventing AD associated dementia. ADNC criteria for resistance and resilience are not well-defined, so we developed stringent pathologic cutoffs for non-demented subjects to eliminate cases of borderline pathology. We identified 14 resistant (85+ years old, non-demented, Braak stage ≤ III, CERAD absent) and 7 resilient (non-demented, Braak stage VI, CERAD frequent) individuals out of 684 autopsies from the Adult Changes in Thought study, a long-standing community-based cohort. We matched each resistant or resilient subject to a subject with dementia and severe ADNC (Braak stage VI, CERAD frequent) by age, sex, year of death, and post-mortem interval. We expanded the neuropathologic evaluation to include quantitative approaches to assess neuropathology and found that resilient participants had lower neocortical pTau burden despite fulfilling criteria for Braak stage VI. Moreover, limbic-predominant age-related TDP-43 encephalopathy neuropathologic change (LATE-NC) was robustly associated with clinical dementia and was more prevalent in cases with high pTau burden, supporting the notion that resilience to ADNC may depend, in part, on resistance to pTDP-43 pathology. To probe for interactions between tau and TDP-43, we developed a *C. elegans* model of combined human (h) Tau and TDP-43 proteotoxicity, which exhibited a severe degenerative phenotype most compatible with a synergistic, rather than simply additive, interaction between hTau and hTDP-43 neurodegeneration. Pathways that underlie this synergy may present novel therapeutic targets for the prevention and treatment of AD.

## Introduction

Alzheimer’s disease neuropathologic change (ADNC) is defined by the presence of extracellular amyloid (A) β plaques with varying levels of intracellular neurofibrillary tangles of hyperphosphorylated tau (pTau) present in well-characterized, stereotypical patterns throughout the brain. In general, the more extensively the brain is involved by Aβ and pTau pathology, the greater the likelihood an individual was cognitively impaired or demented as a result of the clinical manifestations of Alzheimer’s disease (AD). These clinical-neuropathologic correlations have been described in diverse autopsy cohort studies and are summarized in the 2012 NIA-AA guidelines for the neuropathological evaluation of AD [55]. While the vast majority of individuals over the age of 85 at death have some degree of AD neuropathology, those who have at least an intermediate degree of ADNC as defined by the NIA-AA guidelines are considered to have sufficient pathologic burden to explain dementia prior to death. However, there are some individuals with no clinical history of dementia who, at autopsy, are found to have intermediate or even high ADNC; although there is not a widely accepted set of criteria, these individuals may be considered to be resilient to the pathologic changes of AD [5, 8, 23, 62, 82]. Conversely, some people, despite advanced age, do not develop ADNC and can be described as resistant to developing AD [48]. Although under these definitions, resilient and resistant individuals are relatively few in number in autopsy cohorts and represent the extremes of the population, understanding mechanisms that underlie their resilience or resistance to ADNC, which are likely to be distinct, may provide important clues to develop preventive and therapeutic strategies for AD.

Community-based cohort studies have shown that the neuropathologic changes of AD rarely exist in isolation; rather, the majority of elderly individuals are found to have multiple comorbid pathologies at autopsy, most commonly Lewy body disease (LBD), vascular brain injury (VBI) including microvascular brain injury (μVBI), and pTDP-43 pathology, with or without hippocampal sclerosis [14, 19, 35, 41, 43, 63, 78, 85, 98, 99, 101]. The latter has recently been described as limbic-predominant age-related TDP-43 encephalopathy neuropathological change (LATE-NC) defined by a stereotypically distributed TDP-43 proteinopathy in older adults [66]. An active area of research is to determine if or how these diverse pathologies interact, possibly even synergize, to accelerate neurotoxicity and neurodegeneration and eventually functional (cognitive) decline. Individuals with multiple co-morbid pathologies are more likely to experience cognitive symptoms [56, 91]; conversely, those who reach advanced age without cognitive impairment harbor fewer co-existing pathologies on average compared to those with dementia [48, 92]. Therefore, what appears to be cognitive resilience to ADNC may, in part, be due to resistance to developing comorbid neuropathologies. Mechanisms underlying these processes are unknown, but some studies have identified relationships of resilience and resistance to ADNC with systemic exposures and disorders including smoking, cardiovascular disease, diabetes, and medication history [50, 56], as well as lifetime experiences [15, 24, 28, 44, 72, 75, 89], such as educational level, occupation, intelligence, degree of involvement in socially, physically, and cognitively stimulating activities, socioeconomic status, and early life environment.

The concepts of resistance and resilience can be defined and interpreted in many ways, but for the purposes of this study we refer specifically to resistance to the development of and resilience to existing ADNC and associated cognitive impairment. Even with this focused definition of resistance and resilience, we recognize that there are still challenges in applying these definitions in research cohorts given imprecise, qualitative neuropathological criteria for AD regarding pathologic burden and the potential to overclassify resilience and resistance based on less stringent criteria for both pathologic changes and clinical characterization. Therefore, the goal of this study was to rigorously assess both the clinical characteristics and the neuropathology of a group of individuals selected using stringent autopsy and clinical criteria for resistance or resilience specifically to ADNC (Braak stage VI and CERAD frequent) from a community-based autopsy cohort. To do this we not only implemented strict pathologic criteria for ADNC but we also performed a careful review of the rich clinical data available from the Adult Changes in Thought (ACT) study to identify participants who were cognitively intact. This unsurprisingly generated small but relatively homogeneous groups of cognitively intact outliers on either end of the ADNC spectrum which provided the opportunity to identify differences in clinical or demographic variables between groups and compared to cases with ADNC and dementia. We performed an extensive neuropathologic evaluation beyond accepted guidelines for AD and ADRD neuropathologic change, including quantitative measures of pathologic proteins in multiple brain regions and evaluation of synaptic integrity of the perforant pathway, to better characterize the tissue-level findings in these groups compared with matched participants with AD dementia. We hypothesized that non-demented subjects, identified under the most stringent criteria to remove neuropathologically borderline cases, could be discriminated from subjects with AD dementia by clinical and neuropathological traits more specific to resistance and resilience mechanisms which could be further studied in model systems. To that end, based on our findings from this cohort we have developed a novel experimental model of combined proteotoxicity and demonstrated synergistic toxicity of tau and TDP-43 in vivo.

## Materials and methods

### Adult changes in thought (ACT) study cohort

This study was approved by the Group Health (GH)/Kaiser Permanente Washington (KPW) and University of Washington (UW) Human Subjects Review Committees. The Adult Changes in Thought (ACT) study is a population-based prospective cohort study focused on brain aging and risk factors for dementia [47]. ACT is based within KPW (formerly GH), an integrated health care delivery system in Washington state. The ACT study recruits community-dwelling, nondemented adults aged 65 and older from among KP members living across the Seattle, WA area. The original ACT Study cohort included 2581 randomly selected dementia-free older adults enrolled between 1994 and 1996, and an expansion cohort (*n* = 811) was added between 2000 and 2002. In 2005, the study began ongoing enrollment to replace people who die, develop dementia, or drop out. Participants are invited to consider consenting for brain donation but consent is not required to join the study; the brain autopsy consent rate is consistently 25–30%. Cognitive screening, physical function, medical history review, and functional status assessments are administered to ACT participants at study entry and subsequently every two years. Demographic and clinical characteristics are assessed during biennial ACT study visits. Overall comorbidity is measured by self-report using the Charlson Comorbidity Index, which weights comorbid diseases based on risk of mortality [16]. Cognition is measured every two years using the Cognitive Abilities Screening Instrument (CASI) [94]. CASI scores range from 0 to 100, with higher scores indicating better cognition. Scores ≤85 prompt further clinical and psychometric evaluation, and diagnoses of incident dementia are made using standardized (DSM IV and ADRDA) research criteria at a consensus conference [3]. Participants are administratively censored from the ACT cohort upon a dementia diagnosis. Dementia-free participants continue with biennial follow-up.

### ACT neuropathology

A thorough neuropathology evaluation is performed for every ACT participant who provided written consent for research brain autopsy during life [50, 90]. Briefly, all neuropathologic examinations are performed in the UW Division of Neuropathology and the UW AD Research Center (ADRC) Neuropathology Core blind to clinical diagnosis. For decedents with post-mortem interval < 8 h, a rapid autopsy is performed in which more than 60 samples are taken from one hemisphere and flash frozen in liquid nitrogen. The remaining portion of the sampled hemisphere, and the unsampled (intact) contralateral hemisphere (or the whole brain in non-rapid cases) are evaluated for neuropathology and then immersion-fixed in 10% neutral buffered formalin for at least 2 weeks. Following fixation, all brains are evaluated (wholly and after coronal sectioning) for any gross lesions. Tissue samples are dissected from middle frontal gyrus, superior and middle temporal gyri, inferior parietal lobule, anterior cingulate gyrus, primary visual cortex, basal ganglia at the level of the anterior commissure, thalamus, hippocampus at the level of the uncus and lateral geniculate nucleus, amygdala, midbrain including substantia nigra, pons at the level of the locus ceruleus, medulla, and cerebellar hemisphere, processed, and embedded in paraffin prior to sectioning and staining. A microtome is used to cut 4 μm thick tissue sections from formalin fixed paraffin embedded (FFPE) tissue blocks which are stained with hematoxylin and eosin (H&E) and Luxol fast blue (LFB). A Bielschowsky silver stain is manually performed on 8 μm thick tissue sections of mid-frontal and parietal sections. Immunohistochemistry is performed on multiple sections in alignment with the latest guidelines at the time of the autopsy. Primary antibodies used are listed in Table 1. Appropriate positive controls are included for each antibody; negative controls are also run consisting of secondary antibodies of the appropriate species in the absence of primary antibody. All ACT autopsies are evaluated by a Board-Certified neuropathologist for Braak stage, CERAD score, Lewy body disease pathology, hippocampal sclerosis, gross and microscopic (micro) infarcts, frontotemporal lobar degeneration, and all other parameters necessary for a thorough diagnostic neuropathologic exam. Starting in 2012, all ACT autopsies were evaluated according to the NIA-AA guidelines for assessment of ADNC and neuropathology of related disorders [55]; thus, Thal phase scores are not available in routine ACT autopsies that predate 2012. Phospho-TDP-43 (pTDP-43) pathology assessment was not standardized until 2013. Recognizing the challenges of an evolving neuropathology dataset, existing AD neuropathological criteria common to every ACT autopsy (Braak, CERAD) were used for case selection (see below) and then a combined qualitative and quantitative neuropathology evaluation was performed on every case under the same conditions (see below) to better understand differences between resistance/resilience and AD dementia.

**Table 1:** Antibody Characteristics

Target	Clone	Raised in	Poly v. Mono	Company	Dilution	Pretreatment	Brain regions
β-amyloid	6E10	Mouse	Mono	Covance	1:2500	ER2 (20 min)	MFG, MTG, STG, IPL, OCX, striatum, hippocampus, MB, CBL
PHF-tau	AT8	Mouse	Mono	Pierce	1:1000	ER 1 (10 min)	MFG, MTG, STG, IPL, OCX, hippocampus,
α-synuclein	LB509	Mouse	Mono	Invitrogen	1:250	ENZ1 (10 min)	MFG, IPL, amygdala, MB
pTDP-43	ID3	Rat	Mono	Millipore	1:1000	ER 1 (10 min)	MFG, MTG, STG, amygdala, hippocampus
Synaptophysin	MRQ-40	Rabbit	Mono	Novus Biologicals	1:100	ER 2 (20 min)	MFG, MTG, STG, OCX, hippocampus

### Case selection

Cases were selected for this study based on their cognitive status during life and the degree of ADNC as assessed at autopsy. Only neuropathologic data that exists for all ACT autopsies were used for case selection, specifically, Braak stage and CERAD score. The resistant group included all non-demented (CASI within two years of death) ACT participants with Braak stage III or lower and CERAD none who were age 85 years or older. Using these criteria, we identified 14 subjects out of the 684 in the ACT autopsy cohort. The resilient group included all non-demented (CASI within two years of death) ACT participants with neuropathologically verified severe ADNC (defined as Braak stage VI, CERAD frequent). Using these criteria, we identified 7 subjects in the ACT autopsy cohort. AD dementia cases for both resistant and resilient groups were matched in a one-to-one algorithm that linked each index case with same sex, demented (based on clinical evaluation including psychometric testing and consensus agreement) participants with neuropathologically confirmed severe ADNC (Braak stage VI, CERAD frequent). Age at death and year of death were used to identify the best match from the linked set for each case. If more than one matched case was still available, post-mortem interval closest to the index case was used to select the best match, which was then removed from the pool of cases available for the matching algorithm for subsequent cases.

### Standardized Neuropathologic assessment

#### Gross neuropathological exam

As part of the ACT autopsy cohort, every brain (including bilateral cerebrum, cerebellum, and brainstem) had undergone a detailed gross neuropathological analysis including assessment for cerebral cortical atrophy. The degree of atherosclerosis was also noted based on a four-point scale (absent, mild when restricted to branch points in the circle of Willis, moderate when also in other regions at the base of the brain, and severe when present on the convexity of cerebrum). Sampling of bilateral cerebral, brainstem, and cerebellar structures was performed for every case (22 routine samples per brain). Any suspected chronic (cystic) territorial and lacunar infarcts were recorded, measured, and additionally sampled for histopathological examination; the total number of gross infarcts for each case was then recorded. Any other focal lesions, including hemorrhage, mass lesions, and other abnormalities were sampled.

#### Ordinal/semi-quantitative assessment

After resistant, resilient, and AD dementia matched groups were selected based on the existing Braak and CERAD data, we controlled for variations of staining reagents, techniques, and neuropathological interpretation over time by performing an additional and extensive evaluation for neurodegenerative disease on each case based on the 2012 NIA-AA criteria [55]. This involved cutting and staining the original FFPE blocks from diverse regions according to NIA-AA recommendations; additional dissections in wet tissue were not required as the historic routine diagnostic sampling in ACT aligned with the NIA-AA guidelines. We used the newly stained sections to evaluate ADNC for each case; we classified neurofibrillary degeneration by characterizing neurofibrillary tangle distribution across medial temporal, isocortical, and primary sensory cortex by the method of Braak and Braak [11, 12], scored neuritic plaque density in isocortex by the method of the Consortium to Establish a Registry for Alzheimer’s Disease staging [CERAD] [53, 54], and assessed Aβ plaque distribution as described by Thal [95]. pTDP-43 pathology was evaluated in amygdala, hippocampus, temporal and frontal cortex similar to published protocols [66, 101, 103]. We characterized Lewy body disease using routine and immunohistochemical stains in the brainstem, amygdala, anterior cingulate gyrus, and frontal (middle frontal gyrus) and parietal (inferior parietal lobule) cortex according to current guidelines [22] and performed a thorough evaluation for chronic macroscopic and microscopic vascular brain injury. For microvascular brain injury, samples of bilateral middle frontal gyrus, inferior parietal lobule, super/middle temporal gyrus, and medial occipital lobe including calcarine cortex, as well as bilateral neostriatum (at the level of the anterior commissure) and thalamus, were sampled for histologic analysis using hematoxylin and eosin combined with Luxol fast blue (H&E/LFB) stains, and the number of cerebral microinfarcts was recorded for these standardized screening sections based on published protocols [31, 55, 90, 98]. Arteriolosclerosis was assessed on a four-point scale (absent, mild when perivascular hemosiderin was present and walls were slightly thickened, moderate when arteriole walls were markedly thickened, and severe when there was luminal narrowing with prominent lamination (onion-skinning) of the vessel wall).

#### Summary neuropathology score

A summary neuropathology score was created as a descriptive metric to capture the relative magnitude of AD, μVBI, LBD, and limbic-predominant age-related TDP-43 encephalopathy neuropathologic change (LATE-NC) in each individual. Similar to previously published scores [56, 92], this summary neuropathology score derives from the sum of subscores for each of the main axes: (i) ADNC subscore (Braak stage for NFTs expressed as a number and then divided by 2 (0–3) plus CERAD score expressed as a number (0–3), and thus ranging from 0 to 6), (ii) μVBI subscore (number of chronic microinfarcts (CMI) with ≥3 CMI by screening protocol assigned a value of 3 and thus ranging from 0 to 3), (iii) LBD subscore (0 for none, 1 for brainstem LBD, 2 for limbic or amygdala-only LBD, and 3 for isocortical LBD in frontal or parietal cortex), and LATE-NC subscore (0 for none, 1 for amygdala only, 2 for hippocampal, and 3 for isocortical in frontal or temporal cortex).

To extend assessment of ADNC beyond NIA-AA guidelines, the local burden of pTau and Aβ plaque pathology was recorded from multiple brain regions on a three-point scale. All assessments were performed by a neuropathologist blinded to dementia diagnosis and original ADNC classification. Hyperphosphorylated tau (pTau) was evaluated using the AT8 antibody scored as: 0 = no tangles, 1 = rare neurofibrillary tangles (< 3 per 10x field) with sparse neurites, 2 = numerous tangles (3–10 per 10x field) and scattered neurites, and 3 = extensive tangle pathology (> 10 per 10x field) with abundant neurites. Aβ was evaluated using the 6E10 antibody and scored as: 0 = no plaques, 1 = rare plaques (< 5 per 10x field), 2 = numerous plaques (5–20 plaques per 10x field), and 3 = extensive plaques (> 20 per 10x field). Cerebral amyloid angiopathy was graded on a four-point scale (0 = absent, 1 = mild when restricted to the leptomeningeal vessels, 2 = moderate when involving a minority of penetrating arterioles, and 3 = severe when involving a majority of penetrating arterioles) in the frontal, parietal, temporal, and occipital lobes as well as the cerebellum. In addition to assessment of pTDP-43 pathology distribution, in each region a score of 0–6 was assigned based on the density of pTDP-43 pathology (combined inclusions and neurites) in a 20x field (0 = none, 1 = 1–2 inclusions/neurites, 2 = 3–5 inclusions/neurites, 3 = 6–10 inclusions/neurites, 4 = 11–15 inclusions/neurites, 5 = 15–20 inclusions/neurites, and 6 = > 20 inclusions/neurites. Severity of microvacuolation and gliosis, a measure of neurodegenerative change, was assessed in H&E/LFB stained sections on a four-point scale (0 = absent, 1 = mild when limited to superficial cortical layers I and II, 2 = moderate when extending to layers III and IV, and 3 = severe, when extending through all cortical layers to the subcortical white matter) for each brain region examined.

### Quantitative neuropathology using digital slide scanning

Sections immunostained for synaptophysin, pTDP-43, and pTau were scanned using an Aperio ScanScope AT2 (Leica Biosystems Pathology Imaging, Vista, CA) at 20x (0.5 μm/pixel), and stored on a server running Aperio eSlide Manager digital slide repository and database software. Using Aperio ImageScope software (Leica Biosystems Pathology Imaging, Vista, CA), each tissue section was annotated with the freeform pen tool to create regions of interest (ROI) for analysis. Quantitative image analysis of the annotated ROIs was performed using Aperio Brightfield Image Analysis Toolbox software (Leica Biosystems Pathology Imaging, Vista, CA). The RGB color vectors of the blue hematoxylin counterstain (R:0.67, G:0.66, B:0.339) and brown DAB chromogen (R:0.311, G:0.522, B:0.794) were measured using the default Color Deconvolution algorithm in areas where only one of the stain components was present. A third residual component (R:0.02, G:0.999, B:0.02) was used to optimize digital stain separation. The calibrated RGB color vectors were input as parameters into the Color Deconvolution Area Analysis algorithm (pTau stained slides) and IHC Nuclear Quantification algorithm (pTDP-43+ stained slides) to provide digital separation of staining components and allow for quantification of the positive chromogen in the separated DAB channel.

The IHC Nuclear Quantification algorithm was calibrated to count the number of pTDP-43+ inclusions per annotated area. This algorithm finds positively stained objects based on size, shape, and positive intensity threshold parameters. The object segmentation properties were set to use a smoothing radius (2 μm), merging threshold for declustering (2.5), and a manual threshold value (220) of the DAB channel intensity by setting the threshold to the highest possible value and reducing it until background and non-specific staining were removed from the segmented areas. Object shape and size restrictions were set for minimum size (20μm^2^), maximum size (225μm^2^), minimum roundness (0.25), and minimum elongation (0.1). The upper positive intensity threshold for pTDP-43+ objects was calibrated (value 200) by running the above parameters on a training set of 5 positive slides and 5 negative slides. Histogram data of the number of objects per intensity unit was extracted and the cumulative frequencies were used to calculate the final output metric (objects per mm^2^) at each possible threshold cut point. The performance of the algorithm was examined in the positive and negative slides, and the positive threshold was selected at the point where the number of objects per mm^2^ reached a maximum on the positive slides while remained minimum on the negative slides (99th percentile of objects in positive slides, 60th percentile in negative slides). The Color Deconvolution Area Analysis algorithm was calibrated to measure the total amount of staining present in the pTau ROIs. This algorithm counts the number of pixels which exceed an upper intensity threshold in the DAB stain channel. The upper positive intensity threshold (200) was calibrated by measuring the performance of the algorithm on a training set of 5 positive slides and 5 negative slides. Histogram data of the positive area per intensity unit was extracted and the cumulative frequencies were used to calculate the final output metrics (percent positive and average positive optical density) at each possible threshold cut point. The performance of the algorithm was examined in the positive and negative slides, and the positive threshold was selected at the point where the output metrics for the positive slides were greater than the metrics for the negative slides. Some non-specific signal was observed in naturally pigmented lipofuscin and rare hemosiderin regions. All other algorithm values were left as default.

The quantitative analysis data for each ROI were extracted into Microsoft Excel for further analysis. The data included total numbers of pTDP-43+ objects per mm^2^ and for pTau the product of average positive optical density (OD) multiplied by percent of ROI with positive staining (OD*%Positive). Optical density is a measurement of absorbance and is linearly related to the amount of staining present [46]. OD*%Positive is a weighted metric previously used in digitally quantifying immunohistochemical staining [10, 76, 77].

#### Relative Immunointensity ratio

To determine differences in hippocampal integrity, synaptic density in the perforant pathway was measured using synaptophysin-immunostained hippocampal sections. Synaptophysin optical density of the outer molecular layer of the hippocampus was measured in relation to that of the inner molecular layer, generating a relative immunointensity ratio (RIR) similar to previously published methods [80]. ImageJ 1.452j (National Institutes of Health) was used for analysis. Mean pixel intensities of the inner and outer molecular layer of the dentate gyrus just under CA1 were measured. Raw values on an 8-bit scale were normalized to a blank area for each slide. Synaptic RIRs of the outer/inner molecular layer were calculated from (outer molecular layer − blank) / (inner molecular layer − blank) for each case.

### *C. elegans*

#### Strains and transgenics

Wild-type *C. elegans* (Bristol strain N2) was maintained as previously described [13]. Transgenic strains used were CK410 *bkIs410[Psnb-1::hTDP-43* + P*myo-2::dsRED]*, CK491 *bkIs491[Psnb-1::GFP + Pmyo-2::dsRED]*, CK1044 *bkIs1044[Paex-3::tau(WT 4R1N) + Pmyo-2::GFP]* [51, 93].

#### Analysis of transgene segregation and synthetic lethality analysis

Parent *C. elegans* homozygous for human (h) Tau tg (CK1044) but heterozygous for the other transgene of interest (Tg control (CK491) or hTDP-43 tg (CK410)) were generated using a standard mating cross of the two strains, and scoring F2 progeny (F3 animals) for the desired genotype (F2 genotype: htau tg +/+, other transgene +/−). Every progeny from a single htau tg +/+; other tg +/− parent was then singled blind with regards to transgene status onto individual plates with food, and their progeny scored for the transgene of interest (Tg control (CK491) or hTDP-43 tg (CK410). This was performed in triplicate for each transgene of interest.

#### Behavioral analysis

Assessments of *C. elegans* locomotion were carried out as previously described with minor modifications [51]. In brief, 15–20 animals were placed at the center of a 100 mm plate of 5x concentrated peptone nematode growth media uniformly seeded with OP-50 bacteria. Animals were allowed to move freely for 30 min, and the radial distance traveled from the start point was recorded. Distance traveled was converted to micrometers per minute.

#### Immunoblotting

Stage-matched day 1 adult *C. elegans* were harvested and snap frozen. Protein was extracted by resuspending pellets in 1X sample buffer, 20 s sonication, and 5 min boiling. 10 μL of samples were loaded onto precast 4–15% gradient TGX (tris glycine stain-free) gels (Bio-Rad) and electroblotted to PVDF membrane (Bio-Rad). Membranes were blocked in 5% milk; total TDP-43 was detected with mouse monoclonal antibody ab57105 (Abcam) and phosphorylated TDP-43 (pS409/410) was detected with mouse monoclonal antibody TIP-PTD-M01 (CosmoBio). Total tau was detected with Rb17025 antibody (generous gift of Dr. V.M. Lee) and pTau was detected with AT180 (pT231, Thermo Scientific). beta-Tubulin antibody E7 (DSHB) was used as a load control for all samples.

### Analysis/statistics

For demographic and clinical data, binary variables were compared using McNemar’s chi-square test with exact *p*-values, while continuous variables were compared using the Wilcoxon matched-pairs signed-ranks test. We used Wilcoxon matched-pairs signed-ranks tests for analysis of all neuropathology variables. We used Stata (Version 15.1, StataCorp, College Station, Texas) for all analyses. Analysis of genetic distribution and locomotion in *C elegans* experiments was calculated by comparing expected Mendelian genetic ratios for one segregating trait and analyzed by Chi square analysis (GraphPad Prism 7, San Diego, CA).

## Results

### Demographic and clinical characteristics

**Fig. 1:**
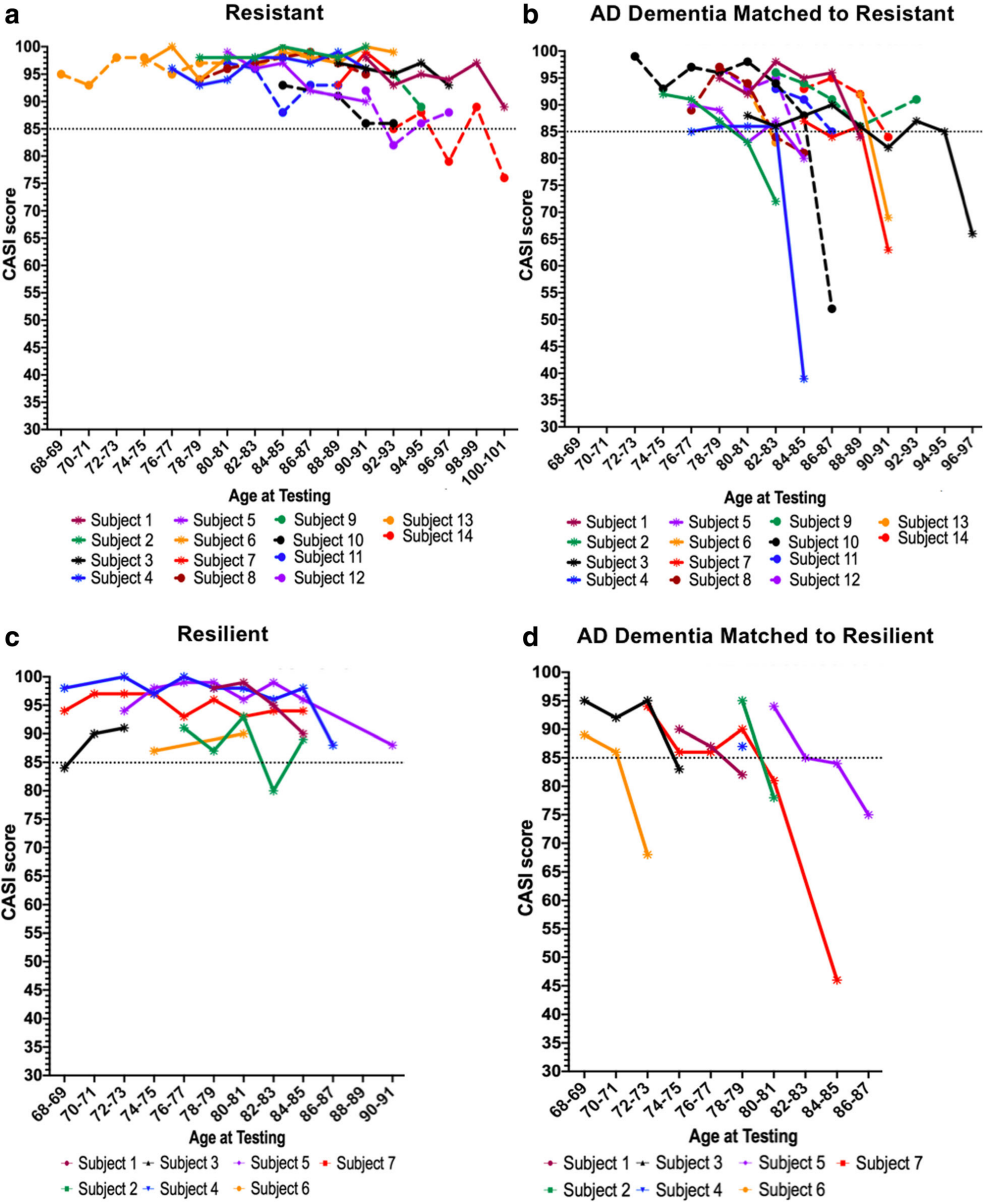
Longitudinal Cognitive Scores. Resistant (**a**) and resilient (**c**) subjects show relatively stable cognitive performance over time. The AD dementia matched subjects (**b** and **d**) were followed until the subjects came to consensus conference (when CASI ≤ 85) and a diagnosis of dementia was made, after which most subjects are no longer administered cognitive testing. Matched pairs are denoted by color and line style

We anticipated our stringent selection criteria would lead to a small number of cases, which was indeed the case. Of the 684 ACT autopsies screened for this study, we identified 14 resistant cases and 7 resilient cases using existing ACT neuropathology criteria. CASI score was used as a primary selection criterion for cases with dementia, and a two-year cutoff was assigned in alignment with prior studies [92, 99]. We then evaluated for each cohort the range of CASI latencies prior to death, with the assumption that a shorter latency provides a higher degree of confidence in the cognitive assessment. We found that the resistant group as a whole had a mean CASI latency of 1.04 years, with a range from 92 days to 1.8 years, and half having a CASI latency of less than one year. The resilient group had a mean CASI latency of 316 days, with a range from 61 days to 1.6 years; with four of the seven cases having a latency of less than one year. Although a final cognitive test within one year of death is desirable, and was achieved in roughly half of the individuals in this study, prior studies have demonstrated the relative reliability of using a two-year cutoff [92, 99]. Most resistant and resilient individuals maintain relatively stable cognitive function as assessed on CASI score across their time involved in the ACT study. Figure 1 illustrates the limited decline in the majority of resistant and resilient subjects over time.

There were not significant differences in most demographics (see Table 2). With respect to genotype, as might be expected, a smaller proportion of the resistant group had ≥1 *APOE* ε4 allele than the matched AD dementia participants (*p* = 0.03), but the proportion of people with ≥1 APOE ε4 allele was not statistically different between resilient participants and their AD dementia matches. The mean number of years of formal education was greater for the resistant group compared to their AD dementia matches (15.1 vs. 13.2 years, respectively), but the difference was not statistically significant (*p* = 0.10). Differences in education levels were not detected between resilient cases and AD dementia matches. These findings are summarized in Table 2.

**Table 2:** Clinical characteristics by resistance and resilience status

	Resistant (*n* = 14)	AD Dementia Matched to Resistant (*n* = 14)	p value	Resilient (*n* = 7)	AD Dementia Matched to Resilient (n = 7)	p value
Age at death (years) mean (SD)	94.7 (3.8)	94.5 (2.5)	0.47	85.2 (6.1)	86.7 (3.5)	0.31
Sex - female n (%)	8 (57.1)	8 (57.1)	1.00	5 (71.4)	5 (71.4)	1.00
Education (years) mean (SD)	15.1 (3.1)	13.2 (2.6)	0.10	15.9 (2.7)	15.4 (3.7)	0.67
*APOE*, n (%) ε2/2 allele	0 (0.0)	2 (14.3)		0 (0.0)	0 (0.0)	
ε2/3 allele	2 (14.3)	0 (0.0)		1 (14.3)	0 (0.0)	
ε3/3 allele	11 (78.6)	5 (35.7)		4 (57.1)	2 (33.3)	
ε2/4 allele	0 (0.0)	0 (0.0)		0 (0.0)	1 (16.7)	
ε3/4 allele	1 (7.1)	7 (50.0)		2 (28.6)	3 (50.0)	
1 copy of ε4 allele n (%)	1 (7.1)	7 (50.0)	**0.03**	2 (28.6)	4 (66.7)	0.50
Age at final study visit (years) mean (SD)	93.1 (3.9)	87.4 (3.7)	**< 0.001**	83.9 (5.6)	79.7 (5.5)	**0.05**
CASI to death (years) mean (SD)	1.0 (0.6)	6.8 (2.8)	**0.001**	0.9 (0.5)	7.0 (3.4)	**0.03**
Charlson Comorbidity Index (cumulative) mean (SD)	2.2 (1.9)	2.1 (2.0)	0.90	3.4 (2.6)	1.1 (1.3)	0.06
Smoking (cumulative pack years) mean (SD)	14.0 (21.1)	14.8 (16.3)	0.43	19.3 (21.6)	4.6 (9.6)	0.15

Wilcoxon matched-pairs signed-ranks test. Note: Cumulative measures were based on self-report at the last ACT study visit attended. Participants diagnosed with dementia were administratively censored from ACT, whereas dementia-free participants continued with biennial study visits. Bold values indicate statistical significance

### Standard neuropathology

#### AD Neuropathologic change

**Fig. 2:**
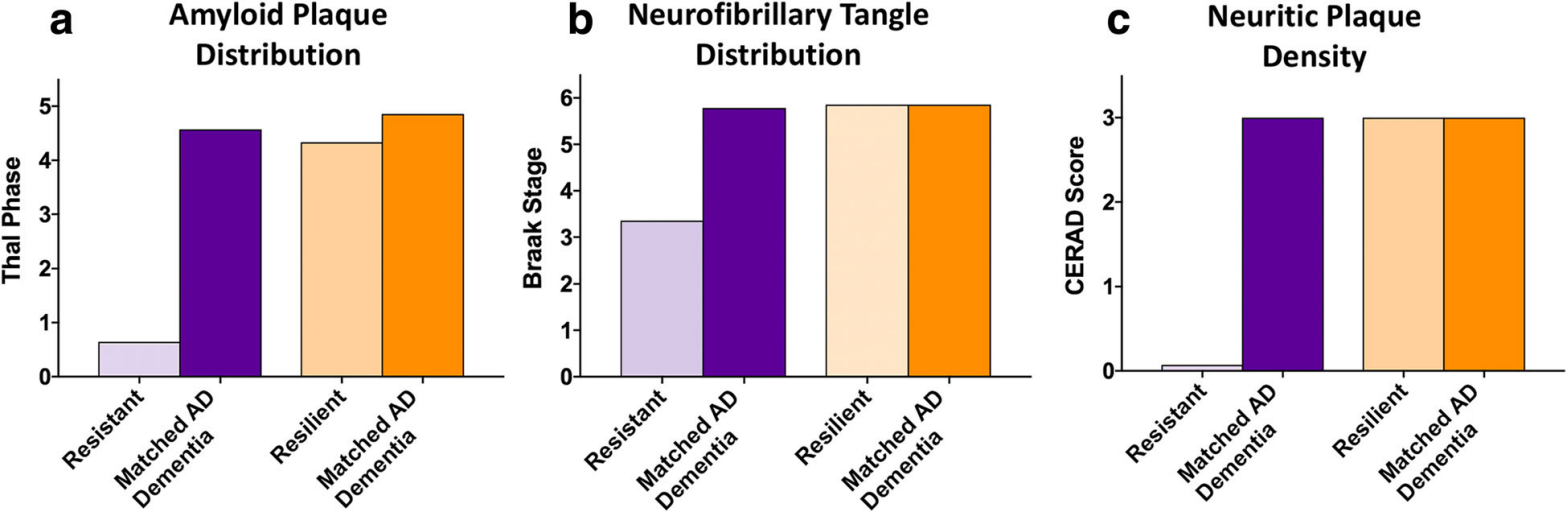
AD Neuropathologic change. Cases were selected based on the degree of AD neuropathologic change. The resistant cases were selected based on a Braak score of III or less and absent neuritic plaques by CERAD criteria. Their AD dementia matches, the resilient cases, and their respective AD dementia matches were selected based on a Braak stage VI and frequent neuritic plaques. Re-evaluation demonstrated the expected distribution of amyloid plaques (**a**), neurofibrillary tangles (**b**), and density of neuritic plaques (**c**), based on these selection criteria

All cases underwent a standardized neuropathologic evaluation using current guidelines and common immunostains, which resulted in reclassification of some cases. Specifically, six cases (one resilient, one AD dementia match for the resilient group, and four AD dementia matches for the resistant group) were re-classified from Braak stage VI to Braak stage V based on the absence of neurofibrillary tangles in calcarine cortex without considering dentate gyrus neurofibrillary tangle burden, which may be less exclusive to Braak stage VI. In the resistant group, seven cases had more widely distributed neurofibrillary tangle pathology using AT8 pTau stains rather than historical (for ACT) Tau2 immunostaining, including some cases with rare cerebral cortex (mostly middle temporal gyrus) tangles and were reclassified (upward) as Braak stage IV [11]. One case in the resistant group was re-classified as a CERAD sparse due to the identification of rare neuritic plaques in the cortex identified by PHF-tau immunostain (AT8). No cases were reclassified more than one level of Braak stage or CERAD score. After reclassification, the resistant group still had lower levels of AD pathology compared to their AD dementia matches (as expected, given the selection criteria), while the resilient group and their AD dementia matches showed similar degrees of ADNC as assessed by Thal phase, Braak stage and CERAD score, verifying that historical misclassification was not an underlying driver of resistance/resilience in this group. These findings are illustrated in Fig. 2.

#### Other Neuropathologic findings

For each case, additional neuropathologic findings were assessed, including the presence and extent of Lewy body disease (LBD) and limbic-predominant age-related TDP-43 encephalopathy (LATE), and evidence of vascular brain injury (VBI), including degree of arteriolosclerosis and atherosclerosis, the number of macro- and microinfarcts, and the severity of cerebral amyloid angiopathy. Overall, the most striking difference between participants without dementia (both resistant and resilient) and their AD dementia matches was the absence of LATE neuropathologic change (LATE-NC) (*p* = 0.0009, resistant and *p* = 0.047, resilient). pTDP-43 pathology was not considered in selecting cases and matches (and wasn’t available for most of the cohort), yet in the resistant group, any pTDP-43 pathology was identified in only 2 of 14 subjects, significantly different from their AD dementia matches where pTDP-43 pathology was present in 13 of 14 cases. In the resilient group a single subject was found to have pTDP-43 pathology, while all but one of the AD dementia matches exhibited pTDP-43 pathology that extended at least to involve the hippocampus. This is remarkable considering pTDP-43 was not part of selection criteria for this study and suggests LATE-NC may be much more prevalent than indicated by historical neuropathological characterization in the ACT study.

We found significant differences between the resistant participants and their AD dementia matches with respect to brain weight (1228 g, 1115 g; *p* = 0.0011), burden of cerebral amyloid angiopathy (0.50, 1.36; *p* = 0.015) and arteriolosclerosis (2.00, 2.57; *p* = 0.031) (for details see Table 3). Atherosclerosis was lower on average in resistant people but this difference was not significant (1.93, 2.29; *p* = 0.097). In the resilient group, chronic macroinfarcts were not identified in resilient subjects but were present in over half of matched AD dementia subjects (0, 0.86; *p* = 0.048). All neuropathology variables are listed, with means, SD, and *p* values, in Table 3. Statistical analysis was not performed for Braak stage, CERAD score, and Thal phase because Braak and CERAD were used as selection criteria, and Thal may be considered directly related to CERAD.

**Table 3:** Neuropathologic characteristics by resistance and resilience status

Pathology (mean+/− SD)	Resistant (n = 14)	AD Dementia Matched to Resistant (n = 14)	p value	Resilient (n = 7)	AD Dementia Matched to Resilient (n = 7)	p value
Brain Weight (grams)	1228 (75)	1115 (84)	**0.001**	1223 (151)	1120 (205)	1.128
Thal Phase	0.64 (0.50)	4.57 (0.51)	ND	4.33 (0.52)	4.86 (0.38)	ND
Braak Stage	3.36 (0.74)	5.79 (0.43)	ND	5.86 (0.38)	5.86 (0.38)	ND
CERAD score	0.07 (0.27)	3.00 (0.0)	ND	3.00 (0.0)	3.00 (0.0)	ND
Atherosclerosis	1.93 (0.62)	2.29 (0.50)	0.097	1.86 (1.10)	1.57 (0.53)	0.421
Arteriolosclerosis	2.00 (0.68)	2.57 (0.51)	**0.031**	2.14 (0.38)	2.29 (0.76)	0.655
Macroinfarcts	0.50 (0.85)	0.57 (0.76)	0.895	0 (0)	0.86 (1.07)	**0.048**
Microinfarcts	1.07 (1.27)	1.50 (1.23)	0.356	0.57 (1.13)	1.14 (1.07)	0.389
CAA severity (OC)	0.50 (0.85)	1.36 (0.84)	**0.015**	1.86 (1.35)	1.42 (0.79)	0.252
Lewy body distribution	1.00 (1.57)	1.50 (1.83)	0.388	0 (0)	0.57 (1.51)	0.317
LATE-NC	0.21 (0.58)	2.40 (0.94)	**0.0009**	0.43 (1.13)	1.86 (0.90)	**0.047**

Wilcoxon matched-pairs signed-ranks test; ND, Not Done; LATE-NC, limbic-predominant age-related TDP-43 encephalopathy neuropathologic change. Bold values indicate statistical significance

#### Summary neuropathology score

**Fig. 3:**
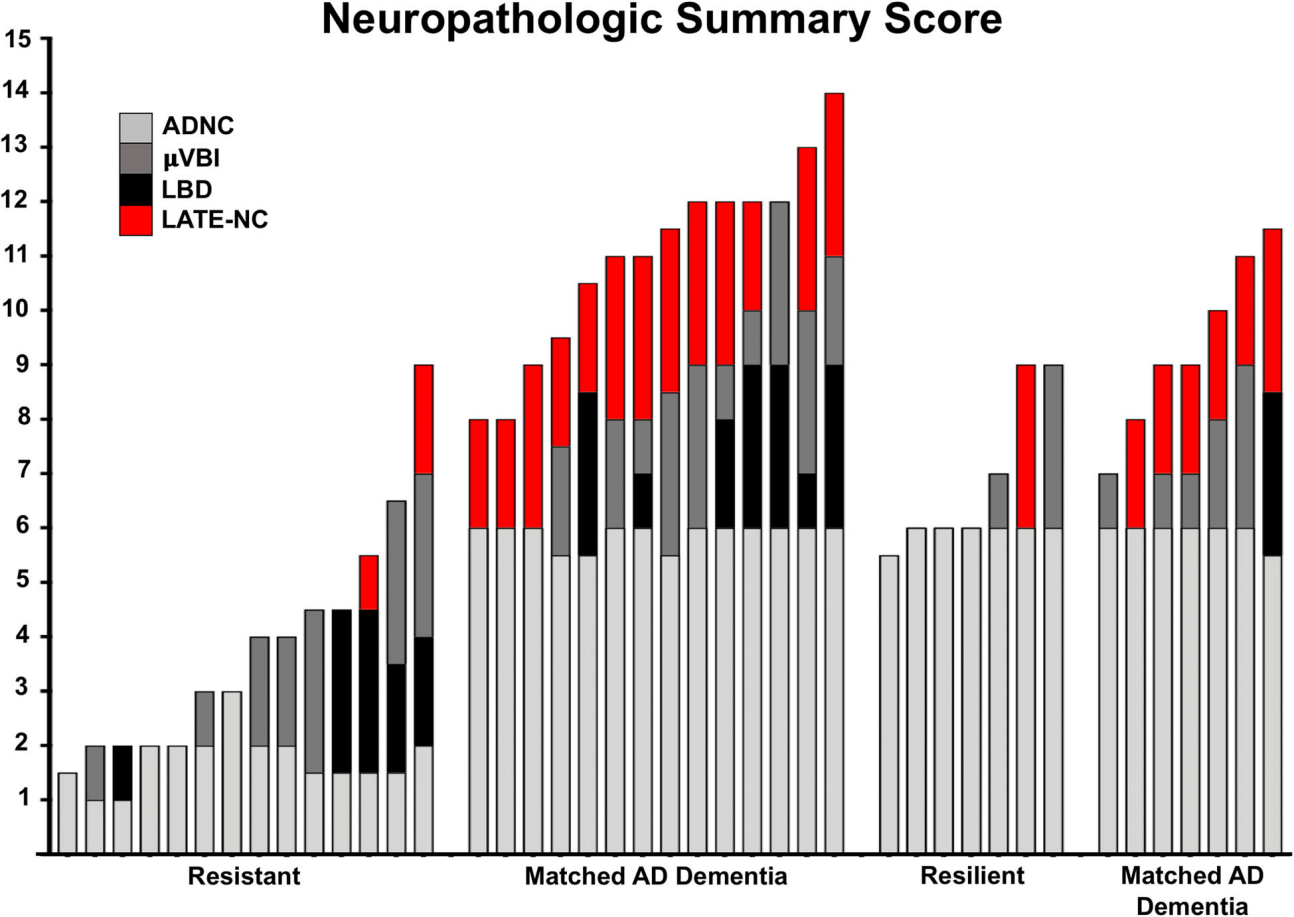
Neuropathology Summary Score. Every subject is depicted by a bar, which is subdivided by colors to represent the different types of neuropathology present for each subject. The grey bars represent AD neuropathologic change (ADNC) and are calculated as (Braak stage/2) + CERAD score, such that the maximum score for ADNC is 6. The slate bars represent microvascular brain injury (μVBI), specifically the microinfarct burden, such that 1 microinfarct = 1, 2 microinfarcts = 2, and 3 or more microinfarcts = 3. The black bars represent extent of Lewy body disease (LBD), such that 1 = brainstem only, 2 = limbic/amygdala predominant, 3 = neocortical. The red bars represent limbic-predominant age-related TDP-43 neuropathologic change (LATE-NC), such that 1 = amygdala only, 2 = hippocampal, and 3 = neocortical (beyond medial temporal). Overall, the resistant and resilient groups conspicuously lack LATE-NC, while pTDP-43 pathology is nearly ubiquitously present in subjects with dementia

Summary neuropathology scores were evaluated for all subjects to represent overall AD and ADRD pathologic burden and are represented graphically in Fig. 3. Each subject’s summary neuropathology score is a bar divided into its corresponding subscores for ADNC (grey), μVBI (slate), LBD (black), and LATE-NC (red); these data have been arranged by summary neuropathology score (lowest to highest), then ranked by AD subscore, followed by μVBI subscore, and then by LBD subscore. These results highlight the findings that it is extremely uncommon for non-demented subjects (whether resistant or resilient to AD pathology) to exhibit LATE-NC, and resistant and resilient cases have less co-morbid neuropathology burden compared with matched AD demented subjects.

#### Semi-quantification

**Fig. 4:**
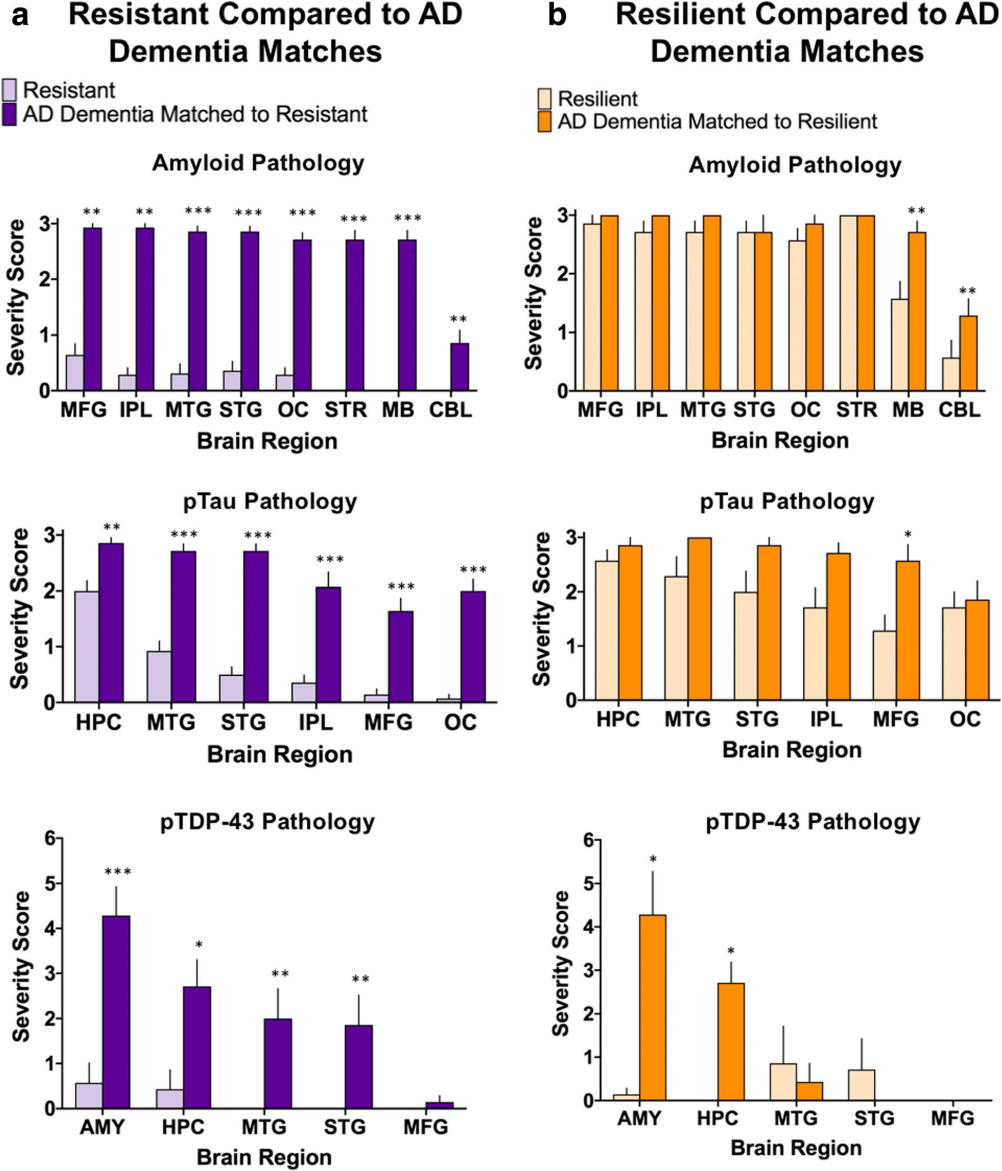
Semi-quantification of pathologic proteins. The burden of each pathologic protein (Aβ, pTau, and pTDP-43) was assessed in each subject by assigning a score of 0–3 for Aβ, 0–3 for pTau and 0–6 for pTDP-43. **a** As expected (based on selection criteria), the resistant group had very little pathology resulting in significant differences for each pathologic protein in nearly every brain region assessed compared to the AD dementia matches. **b** The resilient group showed no significant differences in burden of Aβ pathology throughout the cortex, but did show significantly lower Aβ in midbrain and cerebellum. pTau pathology was significantly less only in the MFG in resilient cases. pTDP-43 pathology showed significantly less pathologic burden in the AMY and HPC. (MFG, middle frontal gyrus, IPL, inferior parietal lobule; MTG, middle temporal gyrus; STG, superior temporal gyrus; OC, occipital cortex; STR, striatum; MB, midbrain; CBL, cerebellum; HPC, hippocampus; AMY, amygdala) ***p < 0.005, **p < 0.01, *p < 0.05; Wilcoxon matched-pairs signed-ranks test

Regional semi-quantitative measurements were made for Aβ (3-point scale), pTau (3-point scale) and pTDP-43 (6-point scale). As expected, there were differences in both Aβ and pTau in resistant individuals compared to AD dementia matches in every brain region assessed (Fig. 4). pTDP-43 was also less abundant in the resistant group in multiple brain regions (amygdala, 0.57, 4.29; *p* = 0.002; hippocampus, 0.43, 2.71; *p* = 0.012; middle temporal gyrus, 0.0, 2.0; *p* = 0.006; superior temporal gyrus, 0.0, 1.86, *p* = 0.009). In the resilient group, we found no difference in cortical Aβ burden compared to AD dementia matches; however, at higher Thal phases the resilient group had less amyloid on average than their dementia matches (midbrain, 1.57, 2.71; *p* = 0.030; cerebellum, 0.57, 1.29; *p* = 0.025). Although resilient cases were matched to AD dementia cases by Braak stage, there was significantly less pTau in the middle frontal gyrus of the resilient group (1.29, 2.57; *p* = 0.037). We also found significantly less pTDP-43 in the amygdala (0.14, 4.29; *p* = 0.028) and the hippocampus (0.0, 2.71; *p* = 0.017). These results are summarized in Fig. 4.

#### Microvacuolation

**Fig. 5:**
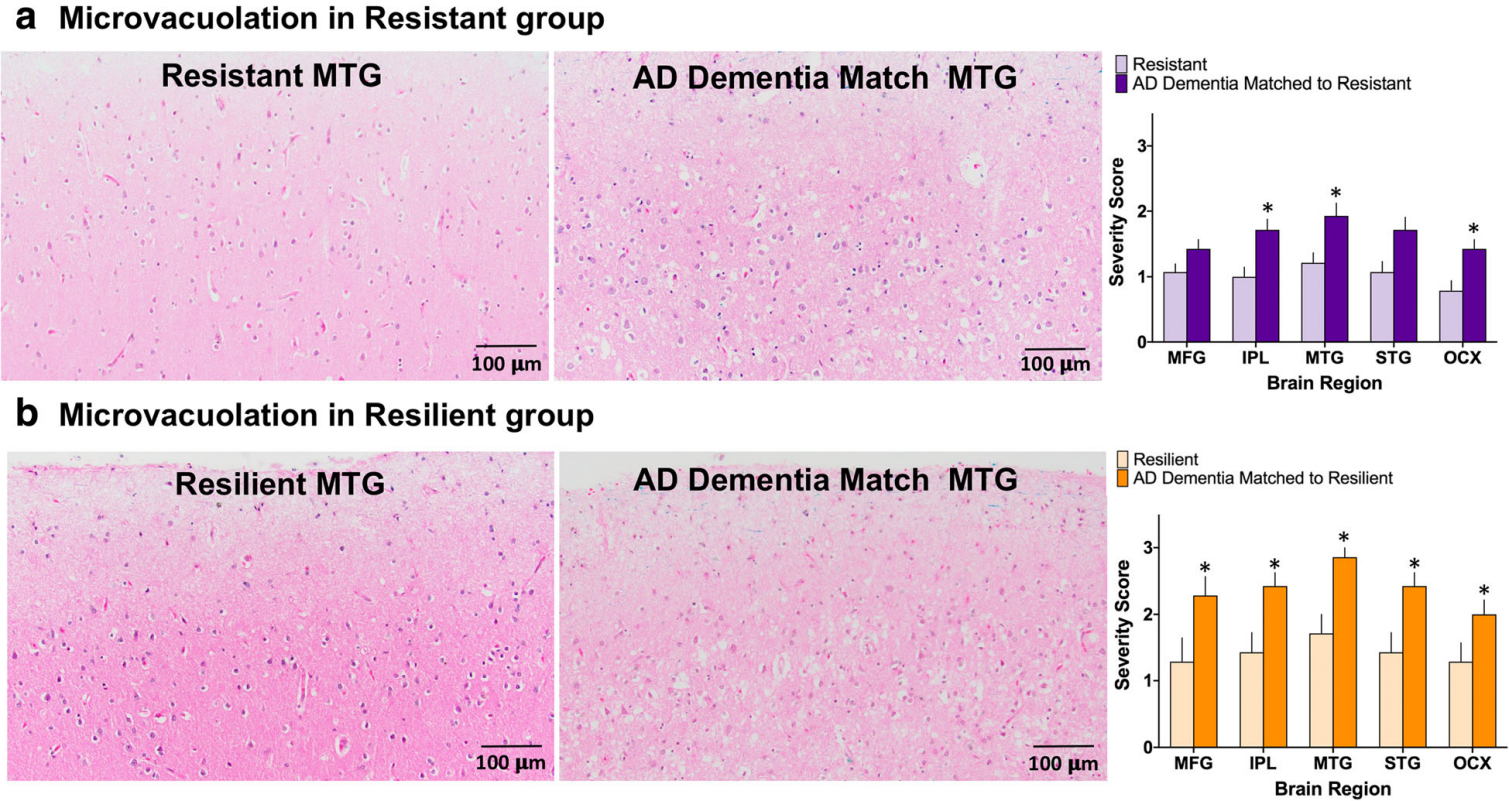
Cortical microvacuolar change. The degree of neurodegenerative tissue damage was assessed in each case on H&E/LFB-stained slides. The microvacuolar changes associated with parenchymal loss and reactive gliosis were scored on a 3-point scale in each neocortical region such that 1 = limited to superficial layers (1–2), 2 = extends to deeper layers (3–4), and 3 = translaminar involvement (5–6). In all AD dementia subjects, the MTG was the most severely affected. **a** Overall there was significantly less parenchymal damage in the resistant group in the IPL, MTG, and OC. There was a trend for less parenchymal damage in the MTG (*p* = 0.0588) and the STG (*p* = 0.0592). **b** Resilient subjects compared to AD dementia matches showed less parenchymal damage in all cortical regions assessed. (MFG, middle frontal gyrus; IPL, inferior parietal lobule; MTG, middle temporal gyrus; STG, superior temporal gyrus; and OCX, occipital cortex). *p < 0.05; Wilcoxon matched-pairs signed-ranks test

In addition to assessing each case for pathologic peptide deposition, we wanted to understand the parenchymal injury associated with dementia, so each cortical lobe in every case was evaluated by H&E/LFB stain for the degree of neurodegenerative change as characterized by the severity of microvacuolation and gliosis. We found significantly less neurodegenerative change in the resistant cases in multiple cortical regions, including inferior parietal lobule (1.00, 1.71; *p* = 0.026), middle temporal gyrus (1.21, 1.93; *p* = 0.037) and occipital cortex (0.79, 1.43; *p* = 0.018) when compared to AD dementia matches. There was also less neurodegenerative change on average in the middle frontal gyrus and superior temporal gyrus, but these differences were not statistically significant (1.07, 1.43; *p* = 0.059 and 1.07, 1.71; p = 0.059, respectively). While these findings may be expected based on selection criteria, we found even stronger relationships of neurodegenerative change and dementia in the resilient group, where every cortical region had significantly less neurodegenerative change than their AD dementia matches (each at *p* < 0.05). These results are summarized in Fig. 5.

### Quantitative neuropathology

#### pTau and pTDP-43 burden

**Fig. 6:**
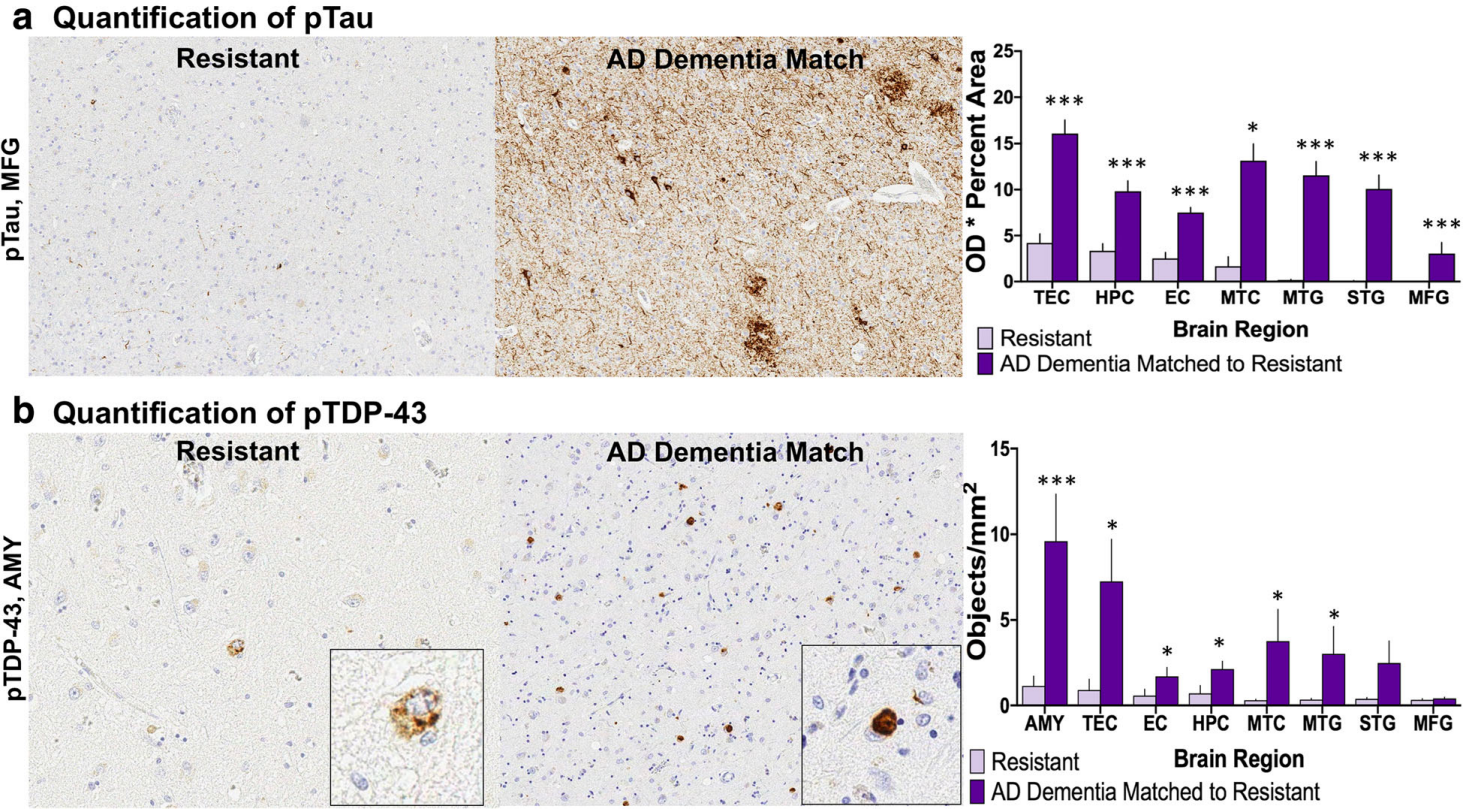
Quantitative pathology for pTau and pTDP-43 in the resistant group. The resistant group had less quantitative pTau in every brain region assessed (**a**) and less quantitative pTDP-43 burden in the majority of brain regions assessed (**b**) compared to the AD dementia matches. (MFG, middle frontal gyrus; MTG, middle temporal gyrus; STG, superior temporal gyrus; MTC, mesial temporal cortex; EC, entorhinal cortex; HPC, hippocampus; TEC, transentorhinal cortex; AMY, amygdala) ***p < 0.005, *p < 0.05; Wilcoxon matched-pairs signed-ranks test

Quantitative analysis of pathologic burden using analysis of imaged immunostained sections was performed for pTau across diverse brain regions, including transentorhinal cortex, hippocampus, entorhinal cortex, mesial temporal cortex, middle temporal gyrus, superior temporal gyrus, and middle frontal gyrus, and for pTDP-43 in amygdala, transentorhinal cortex, entorhinal cortex, hippocampus, mesial temporal cortex, middle temporal gyrus, superior temporal gyrus, and middle frontal gyrus. Analysis of each region of interest (ROI) included a measure of optical density (OD) of the chromogen in positive areas for pTau and a count of positive objects for pTDP-43. As expected, the resistant group had less pTau immunoreactivity on average in all regions assessed compared to the matched AD dementia group (Fig. 6a). There was less pTDP-43 in the resistant group on average in the amygdala (1.15, 9.61; *p* = 0.0030), hippocampus (0.70, 2.13; *p* = 0.016), entorhinal cortex (0.58, 1.70; *p* = 0.048), transentorhinal cortex (0.90. 7.25; *p* = 0.016), mesial temporal cortex (0.31, 3.76; *p* = 0.028), and middle temporal gyrus (0.35, 3.02; *p* = 0.011) (Fig. 6b).

**Fig. 7:**
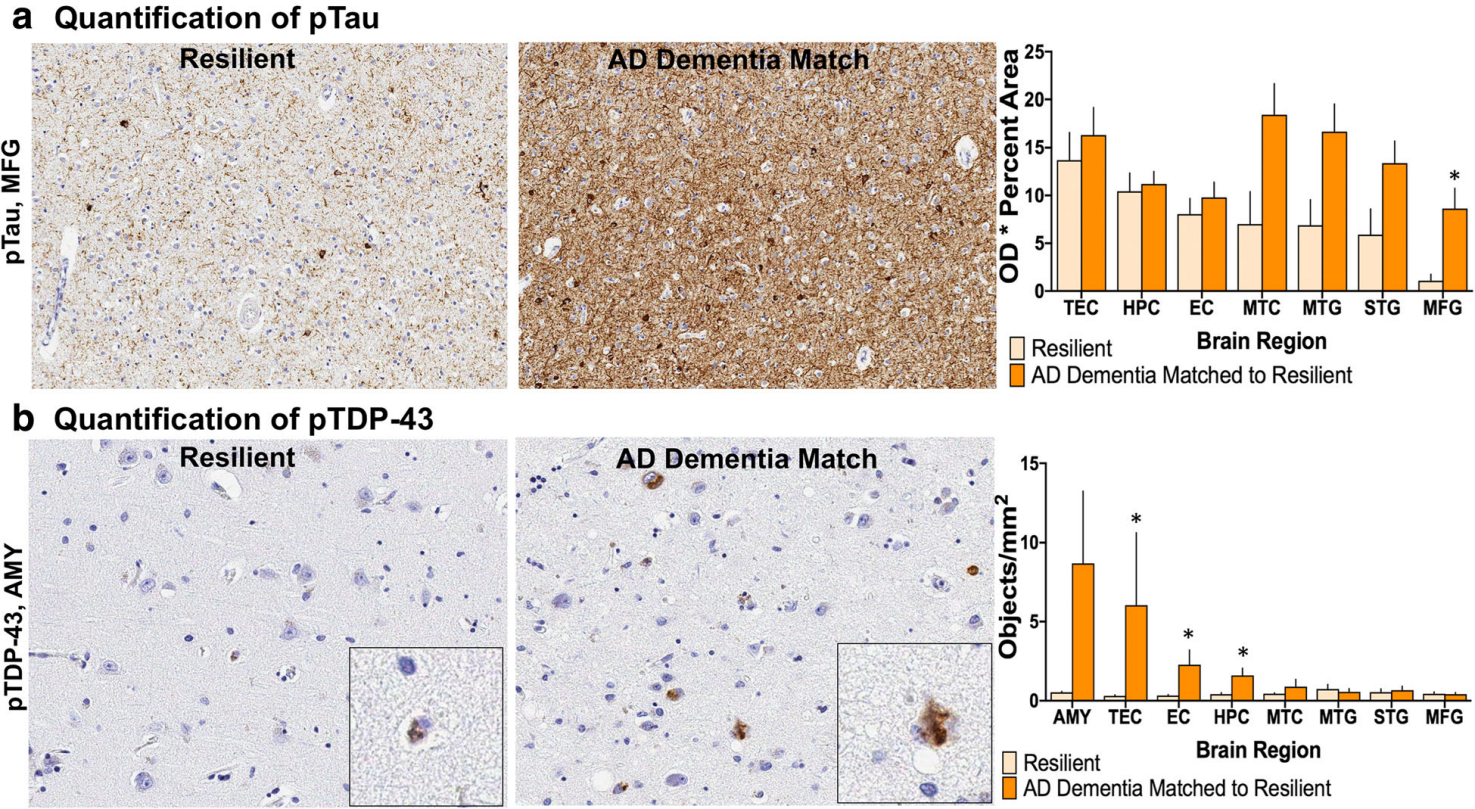
Quantitative pathology for pTau and pTDP-43 in the resilient group: **a** The resilient group had less quantitative pTau burden in the MFG. **b** Quantitative assessments of pTDP-43 also revealed less pathologic burden in TEC, EC, and HPC in the resilient group compared to AD dementia matches. Amygdala showed a trend for reduced TDP-43 immunoreactivity (*p* = 0.0630). (MFG, middle frontal gyrus; MTG, middle temporal gyrus; STG, superior temporal gyrus; MTC, mesial temporal cortex; EC, entorhinal cortex; HPC, hippocampus; TEC, transentorhinal cortex; AMY, amygdala) *p < 0.05; Wilcoxon matched-pairs signed-ranks test

In the resilient group, there was significantly less pTau burden in the middle frontal gyrus on average compared to the matched AD dementia group (1.07, 8.63; *p* = 0.043) but no significant differences were detected in the other brain regions evaluated (Fig. 7a), suggesting the critical point of differentiation between normal cognition and dementia involves pTau burden, at least in part, in frontal cortex. The resilient group had lower levels of pTDP-43 burden on average compared to the matched AD dementia group in the hippocampus (0.42, 1.60; *p* = 0.042), entorhinal cortex (0.33, 2.28; *p* = 0.028), and transentorhinal cortex (0.30, 6.04; *p* = 0.042), while we found no significant differences between resilient and AD dementia matched individuals in amygdala, mesial temporal cortex, middle or superior temporal gyri, or middle frontal gyrus (Fig. 7b).

### Perforant pathway synaptic integrity

**Fig. 8:**
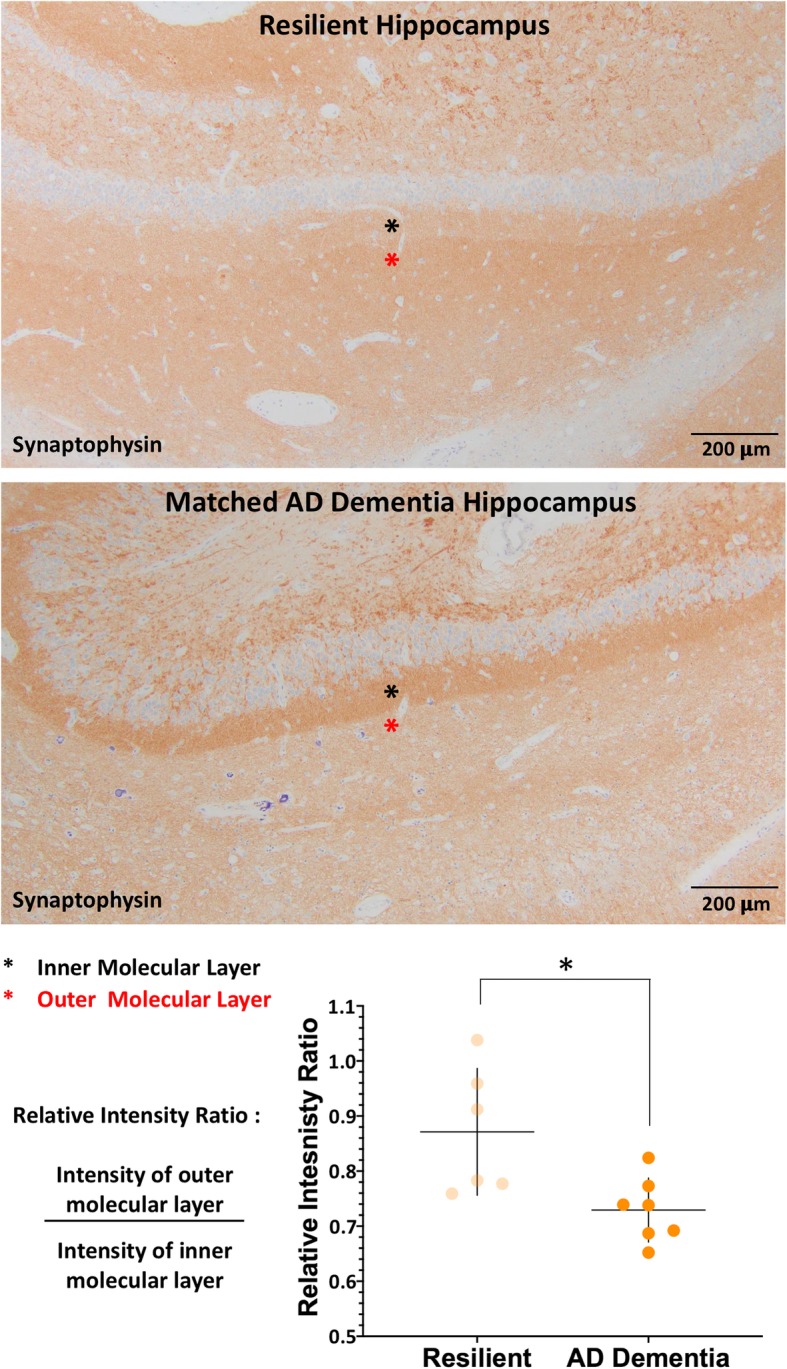
Synaptic integrity of the perforant pathway in the resilient group. The ratio of synaptophysin staining between the outer molecular layer of the dentate gyrus of the hippocampus (red asterisk) and the inner molecular layer (black asterisk) provides a measure of synaptic integrity of the perforant pathway. The AD dementia subjects had a reduced ratio due to a preferential reduction in staining of the outer molecular layer compared to the inner layer while the resilient group maintained a ratio closer to one, reflecting maintained synaptic health. *p < 0.05, Wilcoxon matched-pairs signed-ranks test

Loss of synapses in the perforant pathway has been shown to correlate with cognitive impairment and AD [32, 80]. In particular, synapses within the outer molecular layer of the hippocampal dentate gyrus, which arise from layers two and three of the entorhinal cortex, are affected in AD while the neurons and synapses of the inner molecular layer remain relatively unaffected. To assess the integrity of the perforant pathway synapses we determined the relative immunointensity ratio (RIR) for synaptophysin staining of the inner and outer molecular layers of the dentate gyrus. The resilient group had a mean (SD) RIR of 0.87 (0.12), which was significantly higher than the AD dementia matched group, which had a mean (SD) of 0.73 (0.06, *p* = 0.046) (Fig. 8). There was no difference in mean RIR between the resistant group and their AD dementia matches (data not shown).

#### In vivo modeling of tau and TDP-43 co-pathology

**Fig. 9:**
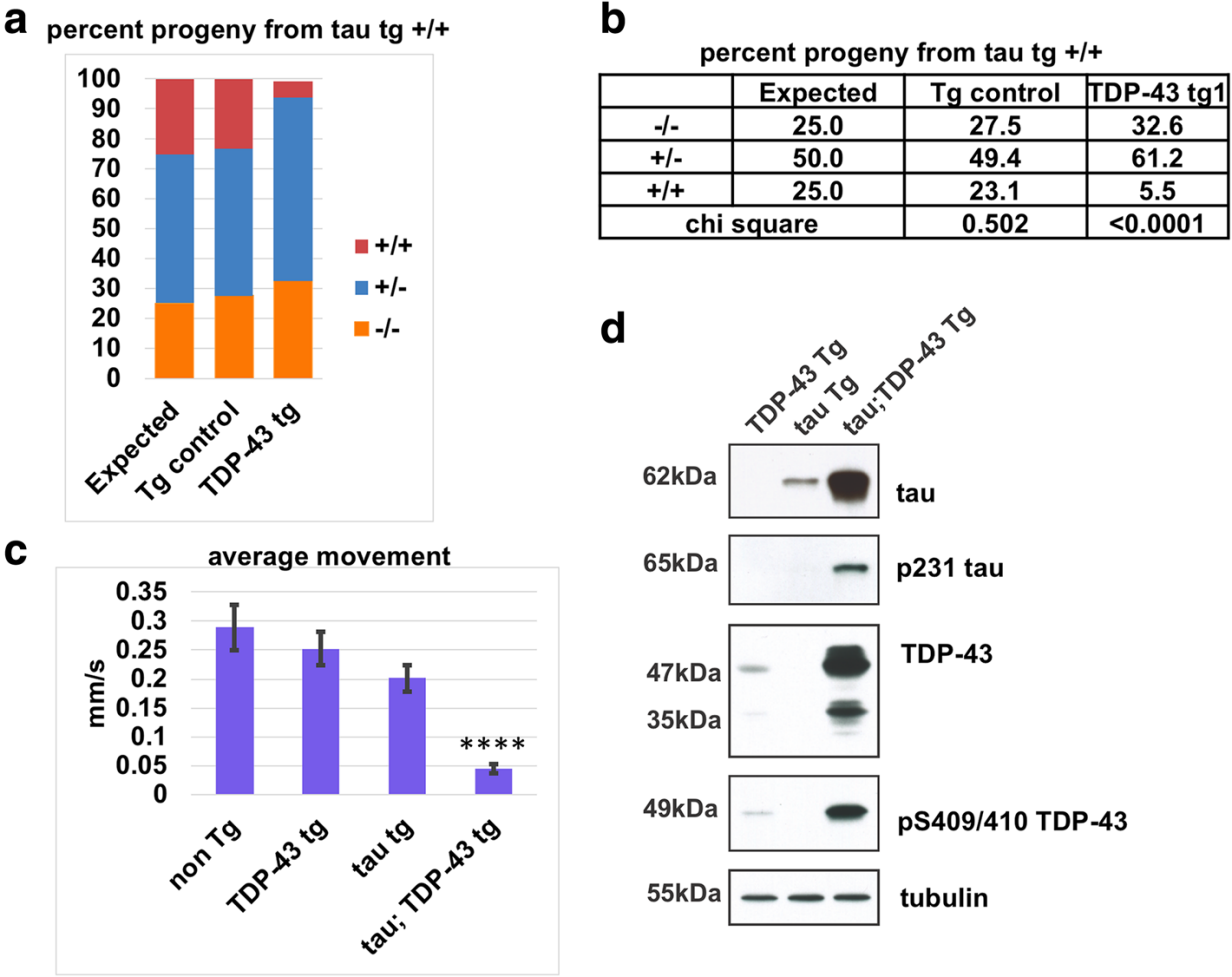
hTau and hTDP-43 synergize in vivo to drive neurotoxicity and protein accumulation. **a**, **b** Pan-neuronal expression of human TDP-43 (hTDP-43) is synthetic lethal with human Tau (hTau) in *C. elegans* transgenic models. Progeny from animals homozygous for hTau tg (+/+) but heterozygous for either GFP Tg or hTDP-43 tg (+/−) were picked blind and then scored for GFP Tg or hTDP-43 tg genotype. Expected Mendelian ratios for assortment of a single genetic element are 25% (+/+), 50% (+/−), and 25% (−/−). Ratios of progeny from animals heterozygous for GFP Tg are not significantly different from Mendelian ratios (*p* = 0.502, Chi square analysis). Ratios of progeny from animals heterozygous for hTDP-43 tg are significantly different from Mendelian ratios (*p* < 0.0001). *N* = 563, GFP Tg. *N* = 561, hTDP-43 tg. **c** Developmentally synchronized L4 larvae of hTau tg (+/+); TDP-43 (+/+) double homozygotes move significantly less than hTau tg (+/+) or hTDP-43 tg (+/+) alone (****p < 0.0005). Statistical significance was determined using one-way ANOVA with Tukey’s multiple-comparison test. **d** Co-expression of hTau and hTDP-43 promotes accumulation and pathological phosphorylation of both proteins in vivo. Developmentally synchronized day 1 adult *C. elegans* were harvested and tested by immunoblot for total tau, phosphorylated tau (AT180), total TDP-43, phosphorylated TDP-43 (phospho-S409/410) and tubulin (load control). Immunoblot shown is representative of three independent replicate experiments

There is a large body of literature associating pTau and pTDP-43 pathology with cognitive decline in AD [2, 6, 35, 41, 60, 61, 66, 77, 101]. We confirmed these associations in this study using highly stringent selection criteria, where higher cortical pTau burden and the presence of pTDP-43 pathology were the key discriminating features between individuals who were resilient to the cognitive impact of AD pathology versus those with clinical dementia. However, it is unknown whether there is a synergistic relationship between pTau and pTDP-43 pathology. To test this, we used transgenic *C. elegans* expressing wild-type 4R1N human tau pan-neuronally (hTau tg) from a chromosomally integrated single, Mendelian segregating singe transgene [93]. hTau tg animals have low levels of tau protein and a very mild behavioral deficit. To test interactions between tau and TDP-43, we crossed hTau tg animals with *C. elegans* expressing wild-type human TDP-43 pan-neuronally (hTDP-43 tg) from a chromosomally integrated single, Mendelian segregating singe transgene [51]. hTDP-43 tg animals have low levels of TDP-43 protein and a very mild locomotion behavior dysfunction as well. To our surprise, we were unable to isolate double transgenic animals with both hTau and hTDP-43 transgenes after picking typical numbers of progeny from a cross, a situation indicative of possible synthetic lethality. To measure any synthetic lethality caused by synergistic toxicity between hTau and hTDP-43 encoding transgenes, we crossed hTau tg and hTDP-43 tg animals and isolated individuals that were homozygous for the hTau tg but heterozygous for the hTDP-43 tg (hTau tg +/+; hTDP-43 tg +/−). We then scored the genotypes of the progeny from these animals for the presence of the hTDP-43 tg (Fig. 9). We found that the genotypes of progeny significantly differed from the expected Mendelian ratios for assortment of a single genetic element (Fig. 9b). As a control, we generated a similar cross between hTau tg and a transgene expressing only GFP pan-neuronally (GFP Tg). Classes of progeny produced from this cross were not significantly different from expected Mendelian ratios (Fig. 9a-b), indicating that toxicity observed is specific for individuals expressing both hTau and hTDP-43. By picking large numbers of progeny from hTau tg +/+; hTDP-43 tg +/− animals, we recovered a rare class of individuals that were homozygous for both hTau and hTDP-43 transgenes (hTau tg +/+; hTDP-43 +/+). In contrast to the hTau or hTDP tg parent animals, these individuals had severe movement dysfunction (Fig. 9c). We then tested whether protein levels of tau or TDP-43 were different in the double transgenic animals. Levels of both total and phosphorylated tau and TDP-43 were dramatically elevated in the hTau tg +/+; hTDP-43 tg +/+ double homozygotes compared to parental single transgenic animals alone (Fig. 9d). Taken together, these data indicate that tau and TDP-43 synergize in vivo exacerbating proteotoxicity.

## Discussion

There is a growing body of literature describing associations with clinical and neuropathological resistance and resilience to AD neuropathologic change (ADNC), but no agreed upon standards for categorization or even nomenclature. We recognized the potential for misclassification using lenient standards for clinical dementia and ADNC and so devised a selection scheme with highly stringent criteria to define resistance (non-demented, Braak <IV, CERAD none, age 85+) and resilience (non-demented, Braak VI, CERAD frequent) to ADNC in subjects with supportive psychometric testing within two years of death. Our goal was to select for cases that would facilitate identification of pathways and pathologies for emphasis in future epidemiological, neuropathological, and translational research. Under these conditions, we identified seven individuals from the ACT study (out of 684 total autopsies) who were cognitively normal within two years of death, and yet at autopsy had severe ADNC, defined as a Braak stage of VI and a CERAD score of frequent neuritic plaques (although standardized assessment using the latest techniques resulted in reclassification of one resilient case to Braak V). By limiting this group to those highest levels of neurofibrillary tangle distribution and neuritic plaque burden, we attempted to exclude variations in Braak stage and CERAD score as drivers of resilience. Similar considerations led us to require that the resistant group died without dementia at age ≥ 85 and had a Braak stage of III or less and a CERAD score of none, ensuring low to no ADNC. These strict definitions created homogeneous, albeit small, groups with respect to ADNC for the purposes of identifying unique clinical characteristics that associated with each group and permitted the pathologic assessment to focus on variables beyond the current guidelines for the evaluation of ADNC.

### Education may play a role in resistance to developing cerebrovascular and AD pathology

After comparing multiple participant characteristics, including years of education, smoking history, and comorbid diseases, we did not identify statistically significant differences between the resistant or resilient groups and their dementia matches. Power to detect differences across these characteristics is low in this stringently-defined group, but there was a trend in the resistant group toward more years of education and toward reduced cerebrovascular pathology (CAA, arteriolosclerosis, and atherosclerosis). Multiple prior studies have demonstrated an inverse association between higher education and cerebrovascular pathology [21, 42, 100]; our results support these prior findings. The literature is mixed on the association between education and AD, with some studies showing a protective effect of education on ADNC and others demonstrating lack of an association [9, 21, 27, 33, 45, 57, 100]. We did not identify an association between educational level and resilient vs. demented matches in this study, but this study may not be powered to detect small effects in this regard; further evaluation in larger samples is warranted.

### *APOE* ε2 and ε3 genotypes are associated with resistance to AD neuropathologic change

We found fewer resistant cases with ≥1 *APOE* ε4 allele; Aβ and pTDP-43 pathology have both been associated with the *APOE* ε4 allele [18, 52, 73, 84, 97, 102], in alignment with our findings. In contrast, resilient cases were not different from AD dementia matches with respect to *APOE* ε4 alleles. This is consistent with prior research on Aβ and *APOE* ε4, since groups in this study were matched based in part on Aβ (CERAD) levels, but does not account for lower levels of pTDP-43 pathology in the resilient group. Given the rich literature associating the *APOE* ε4 allele with increased risk of developing AD, these results were not surprising. We could not control for *APOE* allele frequency in this study due to the small sample size, but it will be important in future larger-scale studies to control for this variable in order to better understand additional mechanisms of resistance to ADNC.

### Cerebrovascular pathology is lower in resilient and resistant groups

Both resistant and resilient groups had lower rates of cerebrovascular pathology on average compared to their dementia matches, although the specific type of cerebrovascular pathology was different across comparisons. Resistant cases had less CAA on average than their dementia matched group, in alignment with the low levels of parenchymal Aβ found in this group. CAA has also been shown to associate with lower cognitive performance independent of the association of CAA on AD pathology [7]. Arteriolosclerosis and atherosclerosis severity was lower on average in resistant cases; interestingly these differences did not translate to fewer macro- or micro-infarcts. Macroscopic infarcts were lower in resilient cases compared with AD dementia matches; vascular brain injury is a common comorbid pathology in late onset AD [17, 25, 49, 85, 91, 99] and macroscopic infarcts have been shown to contribute to cognitive decline and dementia in other cohorts [86]. Thus, one component of the resilience phenotype may include resistance to macroscopic infarcts in the face of severe ADNC, but this effect alone does not entirely explain the resilience phenotype in this group.

### Extent of amyloid pathology may be lower in resilient individuals

There is a debate as to whether assessing the extent of Aβ pathology (Thal phase) in addition to the cortical burden of neuritic plaques (CERAD score) in the evaluation of ADNC provides useful information with respect to ADNC-related neurodegeneration and dementia [48, 74, 87]. For historical reasons, Thal phase was not available as a selection criterion and therefore was prospectively evaluated in every case for this study. Although overall cortical Aβ burden was similar in resilient cases and AD dementia matches, there was greater Aβ plaque burden in regions of higher Thal phases (brainstem, Thal phase 4 and cerebellum, Thal phase 5) in the AD dementia matches, and only two resilient cases had any cerebellar Aβ burden. These results, albeit from a small and highly selected group, support a role for assessment of Aβ distribution in consideration of ADNC to classify cases with respect to dementia to more accurately assess overall ADNC burden. Moreover, this information is critical in determining the impact of Aβ extent and burden in relation to ADNC phenotypic penetrance and, specifically, the functional impacts of Aβ involvement of brainstem and cerebellum. In particular the cerebellum is increasingly recognized for its role in cognition and therefore understanding cerebellar sparing of amyloid pathology may prove to be particularly important in elucidating mechanisms of resilience [34, 83].

### Despite using Braak stage for selection, cortical pTau is significantly lower in resilient cases

We applied stringent ADNC selection criteria to limit the potential for misclassification of cases as resilient due to lower pathologic burden, but even with these criteria, our quantitative algorithms to assess ADNC using digitized slides of immunohistochemically-stained tissue sections still stratified demented and non-demented cases by pathology. This highlights the limitations of current diagnostic guidelines to precisely classify pathologic burden. While several studies have used some form of quantitative or semi-quantitative assessment of pTau burden [58, 68, 79], this approach allowed us to obtain a quantitative measure of proteins of interest from readily available tissue sections that have also been reviewed by standard neuropathologic techniques. Using this technique to measure pTau, we found that resilient cases had lower overall levels of pTau in the middle frontal gyrus on average compared to the matched AD dementia group, supporting the notion that quantitative pTau burden, which includes pTau-positive neurites in addition to neurofibrillary tangles, may predict cognitive decline better than Braak stage. While prior studies suggest this through semi-quantitative assessments of pTau pathology [1, 64, 65], ours is the first study to quantitatively demonstrate lower levels of pTau in the same sections that underwent standard Braak staging and further argues for consideration of both extent and burden of pathologic proteins in AD.

### Limbic-predominant age-related TDP-43 encephalopathy neuropathologic change (LATE-NC) occurs more often in the context of AD dementia than in resilient or resistant groups

We hypothesized that individuals who are either resilient or resistant to ADNC would have less co-morbid pathologies. In addition to assessment of vascular brain injury (described previously), we performed a thorough assessment for AD-associated pathologic proteins (α-synuclein and pTDP-43). While we found no significant differences in Lewy body distribution or α-synuclein pathology between groups with and without dementia, the presence of pTDP-43 pathology was strikingly specific for the AD dementia subjects. Only 2/14 resistant cases had pTDP-43 pathology (compared with 13/14 AD dementia matches) and 1/7 resilient cases exhibited some degree of pTDP-43 pathology (compared with 6/7 AD dementia matches). TDP-43, first identified as the major pathologic protein in sporadic amyotrophic lateral sclerosis (ALS) and the majority of frontotemporal lobar degeneration (FTLD) cases, has only more recently been described in association with AD. Several studies report a high prevalence of pTDP-43 in late-onset AD, reaching up to 50% in some cohorts [2, 30, 60, 96]. The pathologic distribution of pTDP-43 in AD is distinct from that seen in ALS/FTLD, where the neocortex is prominently involved. In AD, pTDP-43 pathology overlaps with that of pTau, demonstrating a predilection for the amygdala and hippocampus and later involving neocortex [4, 38, 39, 59]. Further, neuroimaging-neuropathologic correlations have demonstrated a relationship between pTDP-43 pathology at autopsy and more rapid progression of hippocampal atrophy on antemortem MRI [36, 40]. Individuals who are found at autopsy to have pTDP-43 aggregates are more likely to have been cognitively impaired, as seen in this study, and others have also shown an association between dementia and pTDP-43 pathology in the setting of significant ADNC [40, 41, 60]. Indeed, this age-related pattern of pTDP-43 pathology has recently been considered through a consensus processes organized by NIA, where the term Limbic-predominant, Age-related, TDP-43 Encephalopathy - Neuropathologic Change (LATE-NC) has been assigned to describe the neuropathology of age-related pTDP-43 proteinopathy [66]. In the consensus paper, the authors point out that LATE-NC is often, but not always, associated with ADNC and the relationships between LATE and AD remain incompletely characterized. In this study, the pTDP-43 pathologic findings demonstrate increased frequency of LATE-NC in dementia compared with resistant and resilient cases.

Given the paucity of Aβ pathology in the setting of mild tauopathy, the resistant cases are considered to have either definite (Thal phase 0, *n* = 5) or possible (Thal phase 1, *n* = 9) primary age-related tauopathy (PART) [20]. There is limited literature regarding pTDP-43 pathology in the context of PART but the incidence in our study (14%) is lower than that reported by Josephs et al. [37] in which approximately 29% of PART cases were found to have very low stage of TDP-43 pathology, the majority of which were limited to the amygdala (LATE-NC stage 1). In our study, one resistant subject had LATE-NC stage 1 (amygdala pTDP-43 pathology) while the other had LATE-NC stage 2 (hippocampal pTDP-43 pathology). Both cases had burdensome pTDP-43 deposits, unlike the majority of cases described by Josephs et al., in which often only a single inclusion was identified [37]. Therefore, the lower percentage of pTDP-43 positive cases in this group may in part be due to sampling bias. Regardless, similar to this prior study, we also find no association between pTDP-43 pathology and cognitive status in the resistant group.

The findings in the resilient group are consistent with other reports associating cognitive decline in late-onset AD and the presence of pTDP-43 pathology [2, 26, 35, 40, 60, 66, 97, 101]. To illustrate this association, we highlight two contrasting cases from the resilient cohort, subject 7 and subject 4 (see Fig. 1). Subject 7 is a prime example of resilience, with a high, stable CASI score (94/100 at both the penultimate and final examinations) and significant quantitative pTau pathology in the middle frontal cortex on par with that seen in the matched AD dementia subjects (5.11 compared to 8.63 ± 2.13). Notably, however, this subject lacks pTDP-43 pathology. Therefore, while comparable to the AD dementia group with respect to standard and quantitative assessment of ADNC, a striking discriminating feature in this case is the lack of LATE-NC. Conversely, Subject 4 has slightly lower levels of pTau burden in the middle frontal gyrus compared to the matched AD dementia group (1.22 compared to 8.63 ± 2.13), but did have LATE-NC and a decline in CASI from the penultimate (98) to the final (88) examinations. This individual did not meet criteria for a diagnosis of dementia but did demonstrate a decline in their cognitive function in the setting of combined ADNC and LATE-NC. Given the limited nature of the groups, these findings cannot be used to generate broad conclusions about causation or extrapolate to the larger cohort, however they do suggest an association between LATE-NC and cognitive decline in the setting of ADNC.

Of note, while we did not specifically assess for the neuropathologic subtype of AD (typical, limbic-predominant, and hippocampal sparing) as described by Murray et al. [58], based on the quantitative pTau data, the vast majority of the cases would likely be considered limbic-predominant given the increased optical density of pTau staining in hippocampus compared to the middle frontal gyrus (24 out of 28 cases with severe ADNC had a hippocampus/MFG tau optical density ratio of > 1.5). The remaining four cases had more typical AD pathology with slightly greater optical density pTau staining in MFG than hippocampus, and there were no cases of hippocampal-sparing ADNC. These data suggest that the association between pTau and pTDP-43 is important in the limbic-predominant subtype but given the small number, associations between pTau and pTDP-43 cannot be assessed in other subtypes of ADNC in this study. Although limited, the available literature on this topic has shown that associations between cognitive decline and pTDP-43 are strong in the limbic-predominant subtype of AD but are lacking in the other subtypes [81]. As a single case in point, there was one subject in the AD dementia group matched to the resistance group who had typical AD pathology instead of limbic-predominant, and this was the one subject that lacked pTDP-43 pathology in this cohort. Additional studies are warranted to determine whether distinct pathways are at play for the different pathologic subtypes of ADNC and if the role of pTDP-43 is of particular importance for limbic-predominant ADNC.

We also note the segregation of vascular brain injury (VBI) pathology in AD dementia subjects compared with resilient and resistant groups, which may suggest a relationship between VBI and pTDP43. Thus, the near complete segregation of LATE-NC with dementia strongly supports the notion that resilience to ADNC is in part due to resistance to pTDP-43 pathology, and this may be related to mechanisms of VBI as has been previously postulated, particularly with respect to pTDP-43 and hippocampal sclerosis [2, 69–71]. Future studies modeling this interaction are needed to further understand the association.

### Both resistant and resilient groups demonstrate lack of tissue damage on routine histologic evaluation

In an attempt to compare ADNC with cerebral cortical injury, we evaluated cerebral cortex neurodegeneration from H&E/LFB-stained sections adjacent to those stained for pathologic peptides by assessing the degree of microvacuolation of the cortex, believed to represent parenchymal loss and associated reactive gliosis. In both resistant and resilient groups there was less tissue damage compared to their matched AD dementia groups. This discrepancy was most prominent in temporal cortex. Given the lack of pathology present in the resistant group this finding was not surprising. Many people in the resilient group had substantial Aβ pathology in cortical regions without evidence of prominent microvacuolation, but as a group showed reduced levels of pTau burden and a lack of pTDP-43 pathology. This data, in combination with similar findings (with respect to pTDP-43 and pTau) in the resistant group, suggests that these pathologic proteins may be specifically associated and perhaps interact to promote neurodegenerative tissue changes. Because this is a cross-sectional autopsy study, we cannot determine whether the association between microvacuolation and pTau and pTDP-43 pathology is a cause, consequence, or coincidence.

### Synaptic integrity of the perforant pathway is maintained in resilient individuals relative to those with AD dementia

The hippocampus is involved in the earliest stages of supratentorial pTau pathology in the progression of AD, and is an early site of pTDP-43 pathology that correlates with cognitive symptoms [35, 36, 56]. Hippocampal perforant pathway synapse loss is believed to be an early change in the progression of AD so we set out to compare perforant pathway synaptic injury between resilient/resistant groups and AD dementia matches following an approach previously published by Robinson et al. [71]. We found significantly increased synaptic staining in the outer molecular layer of the hippocampus of participants in the resilient group compared to the AD dementia matched group, but this relationship was not apparent in comparing resistant cases and matched AD dementia subjects. Thus, while tempting to attribute increased synaptic staining to preserved synaptic integrity as a possible basis for the resilience phenotype, this relationship is likely to be more complicated. Given the small sample size, follow-up assessment of the larger ACT cohort is necessary, as well as experimental studies, in order to better understand these pathways and determine specific interactions between pathologic peptides and neuronal and synaptic function and toxicity.

### TDP-43 and tau may interact synergistically to increase the risk of dementia in elderly individuals

Studies in stringently selected human cohorts of resistance and resilience and carefully matched demented subjects with severe ADNC revealed significant differences in cortical pTau burden and the presence of pTDP-43 pathology (LATE-NC). Hyperphosphorylated tau has long been described as a diagnostic pathologic feature of AD, and its distribution throughout the brain (Braak stage) correlates well with cognitive impairment [29, 64, 67]. However, using quantitative methods, we show that despite having the same Braak stage as their matched AD dementia group, the resilient group had less pTau burden on average in cortical regions. The resilient group also tended to lack LATE-NC, and although the mechanism is currently unknown, this study and others suggest that pTDP-43 together with pTau is associated with increased risk of dementia in elderly people [18]. While other studies have shown that the incidence of concomitant pTDP-43 pathology increases with increased Braak stage [35, 36, 38, 88], this study is the first to use quantitative methods to show that higher pTau burden is associated with LATE-NC. Although this is an intriguing relationship, the underlying mechanism cannot be further elucidated in autopsy tissue. We therefore developed a model in *C. elegans* to test whether there may be more than a summative association between TDP-43 and tau*.* In *C. elegans*, we found that co-expressing human (h) Tau and hTDP-43 in the same animal markedly exacerbates the phenotypes over those caused by either peptide in isolation, including synthetic lethality and severe movement dysfunction, and dramatically increased hTau and hTDP-43 protein accumulation. The combined phenotypes were greater than what would be expected if the interaction were simply additive, suggesting a toxic synergism between hTau and hTDP-43. Biochemical assays show that there is more pathological phosphorylated hTDP-43 and hTau present in worms with combined expression than when either protein is expressed alone, further supporting synergistic toxicity between the two peptides that might ultimately explain the strong association of pTDP-43 pathology and dementia with respect to both resistance and resilience. Future studies using this novel tool to study interactions between hTDP-43 and hTau in vivo, and development of vertebrate animal and human cell culture experimental systems, will be critical to better understanding mechanisms of neurodegeneration that exceed resistance and resilience mechanisms resulting in ADNC-related dementia.

### Limitations

By design, dementia-free ACT participants report diseases and health behaviors more proximal to death than demented participants. No difference was detected in Charlson Comorbidity Index between the resistant group and their AD dementia matches. This suggests the resistant group might have maintained better health into older age compared to the demented subjects, since their Charlson Index less than two years before death was similar to the demented group nearly seven years before death. Future studies could be designed to compare these differences directly.

## Conclusions

These results should be considered preliminary; the groups were stringently selected to be informative but as a result were quite small. However, this study is the first to extensively characterize two cognitively intact groups, specifically defined as resilient or resistant based on the presence or absence of severe ADNC and the first to describe LATE-NC in the context of resistance and resilience to ADNC. It is also the first to model combined hTau and hTDP-43 in *C. elegans* and demonstrate the synergistic proteotoxic effects. Because the study utilizes a highly selected cohort of ADNC outliers, additional work must extend these neuropathologic approaches to larger longitudinal cohort studies, including the entirety of the ACT autopsy cohort, as well as address the underlying pathways of tau and TDP-43 synergism in the context of late-onset AD to elucidate novel pathways for the exploration of treatment targets for AD.

## Data Availability

The datasets used and/or analysed during the current study are available from the corresponding author on reasonable request.

## Abbreviations

ACT: Adult Changes in Thought
AD: Alzheimer’s Disease
ADNC: Alzheimer’s Disease Neuropathologic Change
ADRC: Alzheimer’s Disease Research Center
ADRD: Alzheimer’s Disease and Related Dementias
ALS: amyotrophic lateral sclerosis
Aβ: amyloid β
CAA: cerebral amyloid angiopathy
CASI: Cognitive Abilities Screening Instrument
CERAD: Consortium to Establish a Registry for Alzheimer’s Disease
FFPE: Formalin fixed paraffin embedded
FTLD: Frontotemporal lobar degeneration
GH: Group Health
H&E: Hematoxylin and eosin
IHC: Immunohistochemistry
KPW: Kaiser Permanente Washington
LATE: Limbic-predominant age-related TDP-43 encephalopathy
LATE-NC: Limbic-predominant age-related TDP-43 encephalopathy neuropathologic change
LBD: Lewy Body disease
LFB: Luxol fast blue
NFT: Neurofibrillary tangle
NIA-AA: National Institute on Aging-Alzheimer’s Association
OD: Optical density
RIR: Relative intensity ratio
ROI: Region of interest
SD: Standard deviation
TDP-43: Transactive response DNA binding protein 43 kDa
UW: University of Washington
VBI: Vascular brain injury
μVBI: Microvascular brain injury
